# Extracts from Minutes of Atlanta Academy of Medicine

**Published:** 1871-05

**Authors:** 


### *Extracts from Minutes of Atlanta Academy of Medicine.*

---

MARCH 10, 1871.

Reports of cases being in order.

Dr. Alexander desired the opinion of members, as to the probability of labor being retarded by a naturally short cord, or when twined around the child. He had seen cases of the kind seemingly delayed.

Dr. Withers thought the progress of labor is not materially interfered with by this circumstance. He is of opinion, however, that the position of the child may be changed from this cause, and thus indirectly influence the ready delivery.

Dr. Boring thinks the expulsion may be directly retarded by short cord.

Dr. Harden could see no good reason for supposing the expulsion can be interfered with in this way.

Dr. Lowe advocated the affirmative of this question.

Dr. Love read extracts from a report on treatment of burns with carron oil and carbolic acid—forty parts of the former to one of the latter.

He also alluded to other dissimilar cases, in which this liniment was used satisfactorily. The application is made by saturating cotton, and applying it to the denuded surface. Without removing the cotton, it may be kept moist by an occasional application of the mixture. He had found that in the use of this application, the usual constitutional symptoms did not supervene; and his explanation is that the elements which would have been burned out from the blood, by cutaneous respiration, was effectually neutralized by burning out with carbolic acid, over lime, on the denuded surface.

Dr. Harden had used this mixture in the treatment of burns, in the proportion of one part of carbolic acid to about fifty of the oil, with highly satisfactory results.

---

MARCH 17, 1871.

Dr. Ingraham reported a case of *Morbus Brightii*, and exhibited, under the microscope, a specimen of the urine. Tube casts, and crystals of uric acid, and triple phosphates, were plainly brought to view.

Dr. Wells desired to hear the experience of members, as to the frequency of this affection in Atlanta.

Dr. W. F. Westmoreland, in response, stated that he had seen only two cases in the space of four years.

Dr. Love alluded to the importance of the microscope in forming correct diagnosis in renal affections, and of deciding upon the curable character of the various forms of *albuminuria*.

Dr. Wells reported a case of *Anæmia*, in which troublesome epistaxis was a prominent difficulty. Hæmorrhage also proceeded from the auditorial meatus. The case had been under the starving treatment of *water cure*. Albumen was discovered in the urine, and the main source of derangement probably existed in the kidneys. The lungs gave evidence of serious disease, from which the patient died in a few days.

Dr. J. G. Westmoreland reported a case of *Coxalgia*, and desired the opinion of members as to the best manner of relieving suffering attendant upon this affection. Do counter irritation and artificial issues prove serviceable in arresting the progress of this disease?

Dr. W. F. Westmoreland thinks such local treatment unnecessary, if not injurious, and that extension is perhaps the only certain means of relief. He mentioned the temporary mode of effecting extension by a weight swinging over the foot of the bed, attached to the limb, so as to keep up constant extension while in bed. As confinement is injurious, he advised a splint invented by Dr. Sayers, which acts well in extending the limb and forcing the head of the femur from pressing in the acetabulum, while the patient takes necessary exercise in the open air.

In answer to a question by Dr. Love, he expressed the opinion that the local use of iodine in this affection is perhaps impracticable.

Dr. J. M. Boring reported a case of *Orchitis* treated satisfactorily by the application of a solution of nitrate of silver—fifteen grains to the ounce of water—to the scrotum. The case was of eight months' standing, with enlargement and tenderness of one testicle.

Dr. W. F. Westmoreland alluded to some experiments made in a hospital in Paris, which seemed to prove all local applications in *Orchitis* equally useless.

Dr. Wells thinks local treatment of little or no benefit—that constitutional means should be relied on.

MARCH 24, 1871.

Dr. Love presented a new instrument of his own invention, for washing out the nares, and treating diseases of the nasal cavities, called a *Nasal Spritz*, with a written description and statement of its uses and advantages; also a paper on remedies applicable, by the same, in nasal diseases.*

Dr. Logan, President, desired to call the attention of the Academy to the injurious effects of *chloral hydrate* in inflammatory diseases of the respiratory organs. Although, as a hypnotic, he had witnessed very great benefit from its use, yet he is thoroughly satisfied, from his observation, that in pneumonia and other inflammatory diseases involving the air passages, the inflammatory action is aggravated by this remedy.

In answer to inquiries as to the proper dose as a hypnotic, he gave, as the usual dose in his practice, about ten grains. He also mentioned the fact that sometimes gastro-enteric irritation followed its use.

Dr. Love alluded to the injurious effects of *chloral*, and suggested that these effects might, in some measure, depend upon the exhalation of *chlorine* by or through the lining membrane of the air passages. If this element is liberated *within* the system in undue amount, rendering it necessary that it should be *exhaled* by the lungs, may not an effect be produced on the lining membrane similar to the known irritating effects of chlorine gas when *inhaled?* He does not advocate the use of *chloral* extensively.

Dr. Wells considers *chloral* an effective anodyne, relieving pain and quieting nervous agitation as with opiate preparations. He therefore uses the remedy to relieve pain when sleep is not the immediate effect desired.

Dr. Boring agreed with Dr. Wells in his opinion of the anodyne properties of *chloral*. He had used it in a case of great suffering, with complete relief, after opiates had failed to arrest the suffering.

Dr. Ray had seen anodyne effects similar to those of chloroform and opium. He had used the remedy in *mania a potu* successfully, producing sleep. He had not observed any irritation produced on the alimentary mucous membrane.

Dr. Boring mentioned a case of puerperal convulsions in which *chloral hydrate* was used successfully in their arrest.

* This paper was presented in the April number of THE ATLANTA MEDICAL AND SURGICAL JOURNAL.

Dr. Lowe desired to know the secondary action of *chloral.*

Dr. Boring reported the result of the treatment adopted in a case of *orchitis*, reported at the last meeting. In a short time after the solution of nitrate of silver was applied, great relief was experienced, and gradual and permanent improvement attended its use.

